# The Relationship Between Knowledge, Attitude, Practice, and Fall Prevention for Childhood in Shanghai, China

**DOI:** 10.3389/fpubh.2022.848122

**Published:** 2022-03-04

**Authors:** Wen-Yi Liu, Tao-Hsin Tung, Yi Zhou, Dan Tong Gu, Han Yi Chen

**Affiliations:** ^1^Department of Health Policy and Management, Bloomberg School of Public Health, Johns Hopkins University, Baltimore, MD, United States; ^2^Shanghai Bluecross Medical Science Institute, Shanghai, China; ^3^Institute for Hospital Management, Tsing Hua University, Shenzhen, China; ^4^Evidence-Based Medicine Center, Taizhou Hospital of Zhejiang Province Affiliated with Wenzhou Medical University, Linhai, China; ^5^Science Research and Information Management Section, Shanghai Pudong New Area Center for Disease Control and Prevention, Shanghai, China; ^6^Pudong Institute of Preventive Medicine, Fudan University, Shanghai, China; ^7^Clinical Research Center, Institute of Otolaryngology, Fudan University Affiliated Eye and ENT Hospital, Shanghai, China

**Keywords:** prevention, childhood fall, parent, attitude, educational intervention

## Abstract

**Background:**

Early childhood fall is a pressing global public health problem and one of the leading causes of child injury. China has a high proportion of children and a high burden of illness from falls. Therefore, educational interventions to prevent childhood fall would be beneficial.

**Methods:**

We used the outcome of knowledge, attitude and practice questionnaire, which was conducted by Pudong New District of Shanghai Municipal Government, to summarize demographic and baseline characteristics grouped by intervention or not, and analyzed descriptive statistics of continuous and categorical variables. A logistic stepwise function model was established to study the influence of different covariables on the degree of injury, and AIC/BIC/AICC was used to select the optimal model. Finally, we carried out single-factor analysis and established a multifactor model by the stepwise function method.

**Results:**

Attitude and actual behavior scores had significant differences. The intervention and control groups had 20.79 ± 3.20 and 20.39 ± 2.89 attitude scores, respectively. Compared to the control group (5.97 ± 1.32), the intervention group had higher actual behavior scores (5.75 ± 1.50). In the univariate analysis results, fathers' education level, mothers' education level, actual behavior and what cares for children had a significant influence on whether children got injured. In multivariate analysis, attitude had a positive influence on whether injured [odds ratio: 1.13 (1.05–1.21), *P* < 0.001].

**Conclusion:**

Educational intervention for children and their guardians can effectively reduce the risk of childhood falls, and changes in behavior and attitude are the result of educational influence. Education of childhood fall prevention can be used as a public health intervention to improve children's health.

## Introduction

Common childhood injuries, such as road traffic injuries, burns, drowning, poisonings, and falls, occur frequently, with unintentional childhood falls being one of the most common injuries that happen to children and an important cause of childhood morbidity and mortality (1–3). Most pediatric fractures are associated with falls and frequently occur in the upper extremities due to their inclination to protect themselves with their arms during a fall (4, 5). Falls in children aged 0–4 can lead to severe traumatic brain injury (6). Since immune status may be affected by surgical stress and nutritional status, injuries caused by falls in early childhood may affect the child's growth and development and cause a significant psychological burden (7, 8). There is a strong correlation between former experience of falling and fear of falling, which means that falls can it can deteriorate the normal psychological development of children (9). In addition, the heavy economic burden caused by medical expenses was a salient social issue. Between 16 and 50 percent of families in the United States have a significant financial burden due to health care for children (10); the economic burden of medical expenses related to injuries caused by falls is also high in Japan (11). The economic burden associated with falls is even more pronounced among children in developing countries, such as high hospitalization rates and low medical standards.

Falls in young children are more likely to be overlooked than falls in the elderly. In the United States, childhood fall are only one of the leading causes of non-fatal injuries between the ages of 0 and 4, and less than one tenth of falls in older adults (12). Children, on the other hand, are the easiest to prevent and intervene in. The prevention of falls in the elderly is limited by age and underlying diseases. However, children, as well as their parents, are more receptive to education and are more likely to benefit from educational interventions to prevent falls.

Therefore, based on an educational intervention study on children's injury conducted by Pudong New District of Shanghai Municipal Government, we plan to use the Knowledge, Attitude/Belief, Practice (KAP) model to explore the effect of intervention on early children's falls and provide a theoretical basis for the prevention of their falls to help the government and relevant departments carry out primary prevention of falls.

## Methods

### Data Collection

The design of this study was based on a KAP questionnaire collected by the Science research and information management section, Shanghai Pudong New District Center for Disease Control and Prevention (SCDC). This survey mainly targets 0–6 years old children and their parents in Pudong New District, Shanghai. The purpose of the survey is to investigate the risk factors related to child injury, and the situation of injury. This trial has been reviewed and approved by the Institutional Review Board (PW2014B-1). All participants and their guardians knew the information and signed the consent form.

### Data Source

Before the investigation, the staff coordinated the investigators of the community health service center and neighborhood committee to conduct centralized training on the baseline investigation to ensure the implementation of the intervention. Then, during the training, they share their experience and exchange the problems that the trainees may encounter in the investigation process.

After accepting the suggestions of the neighborhood committee and the selection of geographical location, the project team randomly selected two kindergartens in Yangjing and Puxing communities (Boshan Kindergarten and Dandelion Kindergarten) to carry out a baseline questionnaire survey, which lasted for 2 months (from December 2014 to January 2015). Parents of children aged 0–4 were randomly selected from the neighborhood committee and kindergartens as the survey objects. From January to February 2015, all questionnaires were subject to quality control, and the questionnaires with illogical problems were reinvestigated. Afterwards, qualified questionnaires were entered uniformly twice by specialists. Finally, after cleaning, sorting and logically verifying the database, the working group statistically analyzed the data and finished the baseline survey report.

### Intervention Design

**Figure 1:**
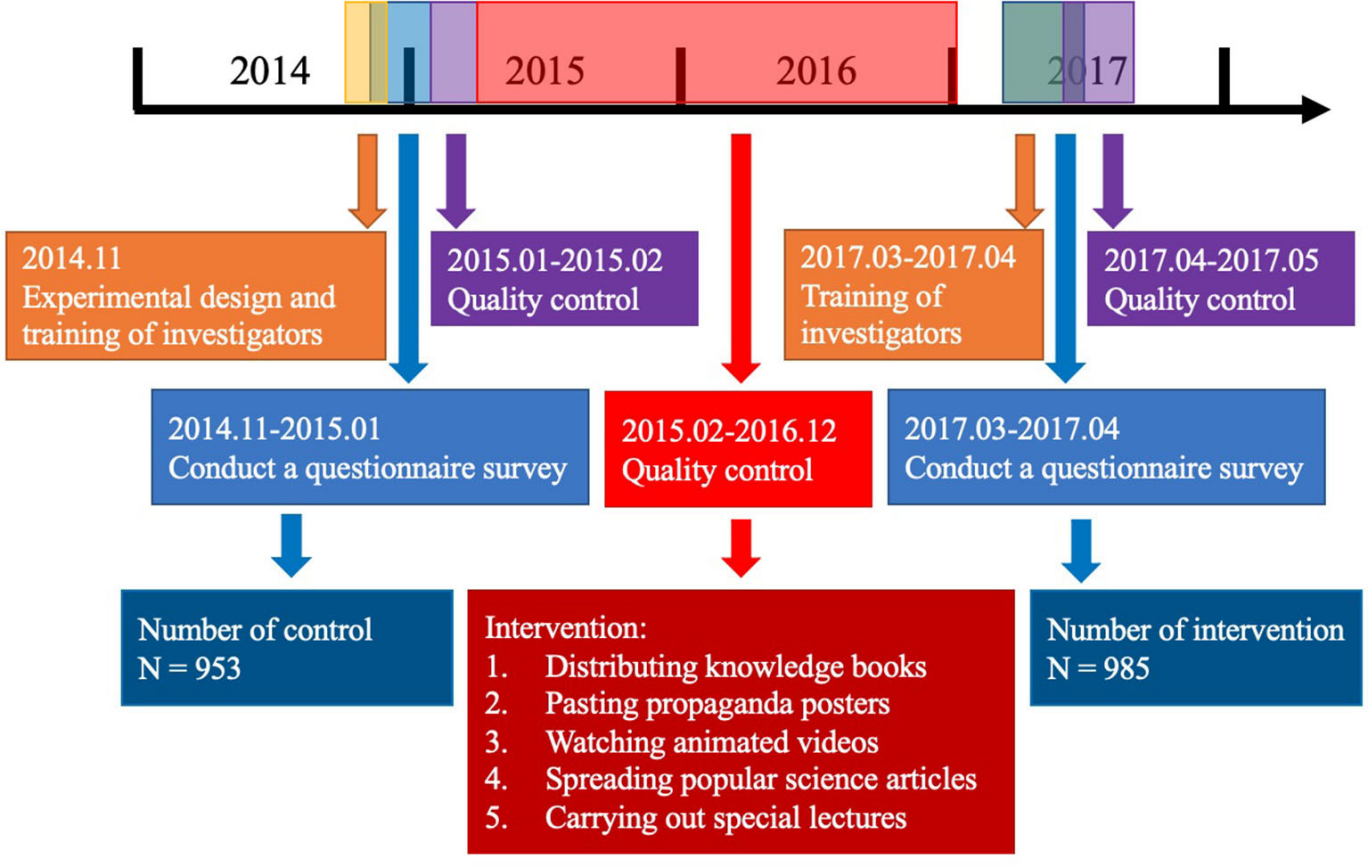
The design of the education intervention.

The entire trial design can be divided into three phases, lasting from November 2014 to May 2017 (Figure 1). The questionnaire we used came from a part of the unified questionnaire on children's injury knowledge, attitude/belief and behavior of the SCDC (see Appendix). The questionnaire was is divided into two parts: basic information and parents' knowledge, attitude/belief and behavior about injury. The basic information includes the child's date of birth, gender, height, weight, parents' occupation and educational background. The second part of the questionnaire was is divided into three parts according to the content of knowledge, beliefs and behavior. The full score of the knowledge part is 7 points. One point will be obtained as long as you fill in the fall in K5; one point will be given when choosing injury as the answer to K6; in K7, one point will be given when boys and girls have the same chance of injury; and one point will be given when other items choose “yes”. The full score of attitudes is 24 points. According to the options, the scores of options 1 to 4 are 4 to 1, respectively. In A7, the questionnaire also provides four open-ended questions to better understand parents' attitudes. The full score of behavior is 43. The first seven questions (P1–P7) are judgment questions (yes or no), with one point for each question; P9–P15 are single choice questions, with five options for each question. P11–P13 are “always” with 5 points, and “never” with 1 point. The scores of other questions are opposite (Never = 5, Always = 1). At the end of the questionnaire, there is a question about the severity of children's injury (F14), which was is used to judge the degree of children's fall injury.

#### Stage 1–Basic Survey

Before the investigation, the staff coordinated the investigators of the community health service center and neighborhood committee to conduct centralized training on the baseline survey to ensure the implementation of the intervention. Experience sharing meetings were organized during the training to help investigators learn, communicate and provide solutions to problems that may be encountered in the investigation process.

#### Stage 2–Intervention

The intervention was conducted from January 2015 to December 2016. It adhered to the injury tertiary prevention principle. The working group mainly delivered health promotion and health education as the primary prevention measure to prevent risk factors for children falling identified during the baseline interview and the baseline survey. Meanwhile, the working group paid special attention to the environmental tactics of injury prevention. They raised community residents' safety awareness of children falling by conducting evaluations of the community environment and helped promote a safer living environment.

The intervention included falling prevention at home (avoiding climbing the furniture, railing the balcony, avoiding jumping on the sofa, etc.), falling prevention at sports (warm up before sports, wear protective accessories, avoid using adult sport equipment, etc.) and falling prevention at schools (avoid chasing in the hallway, climbing the window, caution slipping in the toilet, etc.). Through different health education targeted for children, adults and teachers respectively, the intervention helped raise the entire community's safety awareness to prevent children from falling.

During the course of the intervention, the working group disseminated promotional brochures such as *Children's Home Safety, Winter Break Safety Manual, Intervention Manual for Falling (student version), Intervention Manual for Falling (parent version), Intervention Manual for Falling (teacher version)* several times to the intervened communities and kindergartens. They also posted educational posters with information revolved around prevention from falling high, prevention from athletic injury and prevention from traffic injury on protruding areas of bulletin boards in communities and kindergartens. In addition, they distributed cartoon videos about injury prevention in kindergartens and required children to watch at least once quarterly. Moreover, they also promoted the WeChat subscribed account (a social software widely used in China) of the Pudong CDC among residents. The working group also invited experts on injury prevention to give relevant lectures in the intervened communities in January 2017. The audience was consisted of parents of 0–6-year-olds. The lecture covered topics from basic knowledge of children's injury, high-risk locations of injury, common reasons for children falling, alerts for home safety, road safety, sport safety and walking safety. The lecture was very easy to digest by giving vivid examples to illustrate the key points of children falling prevention, and so was well-received by the audience.

#### Stage 3–Secondary Survey

In March April 2017, the community health service (CHS) and investigators were trained again to conduct a new round of questionnaire surveys in the target kindergartens. Secondary data entry was conducted from April to May 2017 by independent investigators, and all questionnaires were subject to quality control by study personnel. After the database was cleaned, curated, logically reviewed, the data were statistically analyzed, and the return visit results were combined to form the final assessment report.

### Statistical Analysis

All questionnaires were analyzed according to their randomized condition. Demographics and baseline characteristics such as age, gender, BMI, parents' education level, parents' job and KAP scores were summarized by group, which was defined by whether the intervention was performed. We summarized the descriptive statistics of continuous variables (mean, standard deviation) and categorical variables (number and proportions of participants in each category). Differences in characteristics between the groups were tested with chi-square for categorical variables, and paired *t* test was used for continuous variables. We built a logistic model with a stepwise function to determine the influence of different covariates on hurting and used AIC/BIC/AICC to choose the best model. Single-factor analysis was performed first, and a multifactor model was built by a stepwise function. Two-sided values of *p* < 0.05 were deemed statistically significant. Statistical analyses were performed using R software (version 4.1.2).

## Result

Descriptive statistics of the individuals are presented in Table 1. 1,938 out of 2,000 participants completed our study with a 96.9% response rate, 953 of whom were categorized into a control group and 985 individuals in an intervention group. There were more females than males in each group, 51.3 and 51.5% respectively. The average age of control group was lower 2.09 (SD = 1.48) compared with 3.79 (SD = 1.99) in intervention group. There were 73.7% participants in control group and 77.8% in intervention group that were the only child of their families. Intervention group had higher knowledge, attitude and actual behavior scores and lower behavior in mind scores than control group. There were more children (4.3%) sent to hospital due to fall in control group while there were more children reported needing rest due to falling (6.1%) in intervention group.

**Table 1:** Descriptive statistics of the study population.

		**Invention (*n* = 985)**	**Control (*n* = 953)**	***p***
Age [mean (SD)]		3.79 (1.99)	2.09 (1.48)	<0.001
Gender [mean (SD)]	Male	478 (48.5)	464 (48.7)	0.98
	Female	507 (51.5)	489 (51.3)	
BMI [mean (SD)]		16.57 (3.45)	16.46 (2.60)	0.434
Father job (%)	Production staff	58 (5.9)	107 (11.2)	<0.001
	Merchant	223 (22.7)	149 (15.6)	
	Institutional staff	204 (20.7)	259 (27.2)	
	Professional technicians	294 (29.9)	290 (30.4)	
	Other	205 (20.8)	148 (15.5)	
Mother job (%)	Production staff	25 (2.5)	67 (7.0)	<0.001
	Merchant	240 (24.4)	191 (20.0)	
	Institutional staff	217 (22.1)	263 (27.6)	
	Professional technicians	205 (20.9)	194 (20.4)	
	Other	295 (30.0)	238 (25.0)	
Father education level (%)	Junior high school and below	28 (2.8)	21 (2.2)	0.291
	High school/technical school/technical school	324 (32.9)	342 (35.9)	
	Bachelor and above	633 (64.3)	590 (61.9)	
Mother education level (%)	Junior high school and below	45 (4.6)	25 (2.6)	0.028
	High school/technical school/technical school	379 (38.6)	404 (42.4)	
	Bachelor and above	559 (56.9)	524 (55.0)	
Who take care (%)	Parents	380 (38.6)	272 (28.5)	<0.001
	Grandparents	583 (59.2)	651 (68.3)	
	Relative	12 (1.2)	19 (2.0)	
	Nanny	7 (0.7)	11 (1.2)	
	Other	3 (0.3)	0 (0.0)	
Whether only child (%)	Yes	766 (77.8)	702 (73.7)	0.04
	No	219 (22.2)	251 (26.3)	
Knowledge [mean (SD)]		5.74 (1.02)	5.73 (0.92)	0.869
Attitude [mean (SD)]		20.79 (3.20)	20.39 (2.89)	0.005
Behavior actual [mean (SD)]		5.97 (1.32)	5.75 (1.50)	0.001
Behavior mind [mean (SD)]		30.63 (3.54)	30.58 (3.16)	0.733
Behavior [mean (SD)]		36.60 (4.09)	36.34 (3.75)	0.144
Fall to hospital (%)	Yes	42 (4.3)	36 (3.8)	0.668
	No	943 (95.7)	917 (96.2)	
Fall to rest (%)	Yes	60 (6.1)	57 (6.0)	0.995
	No	925 (93.9)	896 (94.0)	

**Figure 2:**
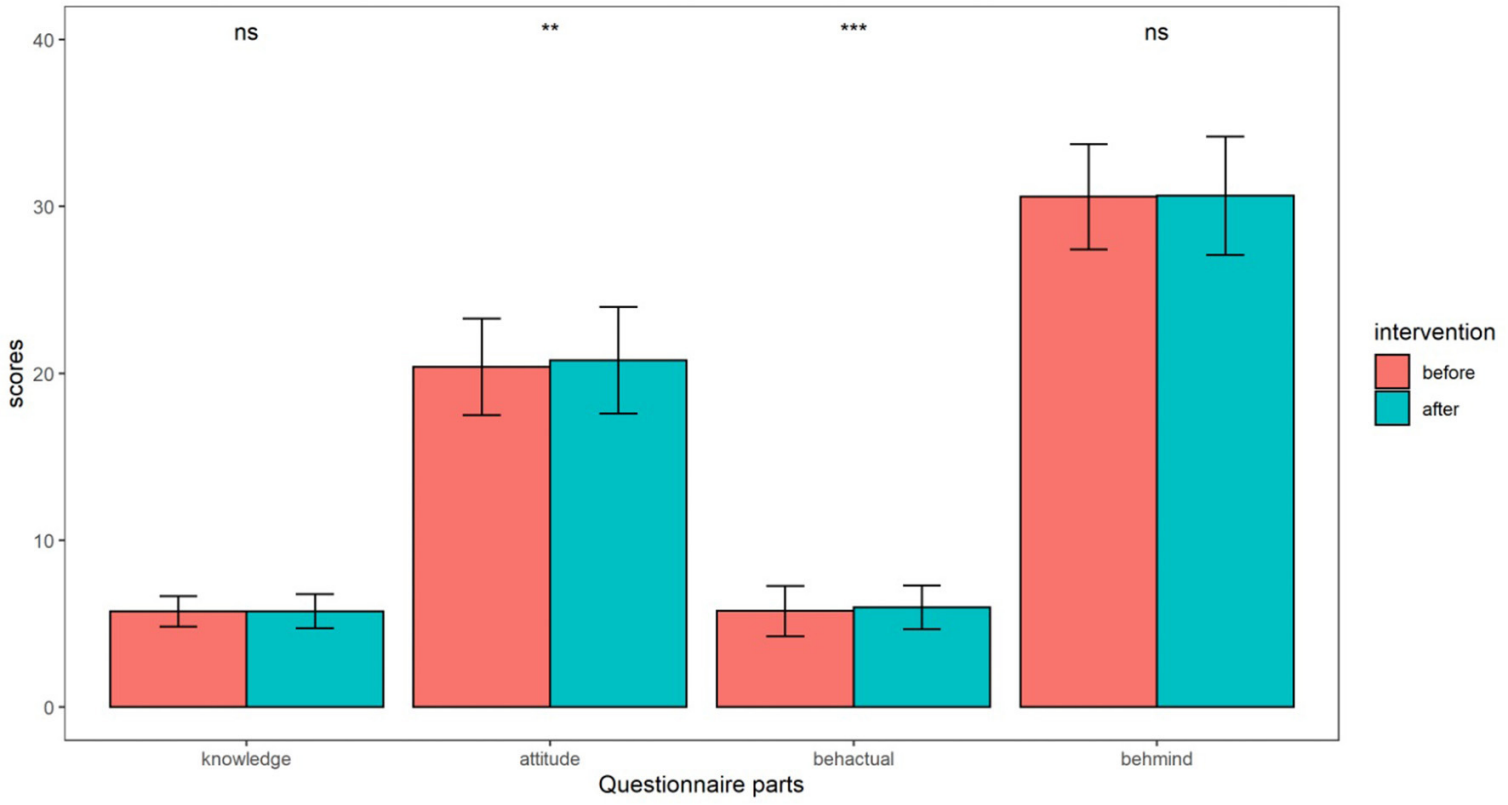
Differences of questionnaire scores in two groups. ns means non-significant; * means <0.05; ** means < 0.01; *** means < 0.001.

Differences in questionnaire scores in the two groups are shown in Figure 2. Attitude and actual behavior scores had significant differences. Intervention group had higher knowledge scores (5.73 ± 1.02) than control group (5.73 ± 0.92). Intervention and control group, respectively had 20.79 ± 3.20 and 20.39 ± 2.89 attitude scores. Compare to control group (5.75 ± 1.50), intervention group had higher actual behavior scores (5.97 ± 1.32). Intervention group had higher mind behavior scores (30.63 ± 3.54) than control group (30.58 ± 3.16).

Table 2 shows the scores of different parts of questionnaire for parents with different severity of falls. Of totally 204 children who were reported hurt, 175 children were mildly injured while 29 children were moderately to severely injured. Parents of moderately to severely injured children had higher attitude scores (21.69 ± 2.00), knowledge scores (5.93 ± 0.70), behavior in mind scores (31.45 ± 2.34) and actual behavior scores (5.68 ± 1.16) than parents of mildly injured children (21.15 ± 2.28), (5.78 ± 0.86), (30.21 ± 3.03), and (5.59 ± 1.43).

**Table 2:** Questionnaire scores for people with different severity of falls.

	**Mild (*n* = 175)**	**Moderate to severe (*n* = 29**	***p***
Knowledge [mean (SD)]	5.78 (0.86)	5.93 (0.70)	0.364
Attitude [mean (SD)]	21.15 (2.28)	21.69 (2.00)	0.23
Behavior actual [mean (SD)]	5.59 (1.43)	5.68 (1.16)	0.755
Behavior mind [mean (SD)]	30.21 (3.03)	31.45 (2.34)	0.037
Behavior [mean (SD)]	35.79 (3.61)	37.11 (2.90)	0.067
Knowledge attitude [mean (SD)]	26.92 (2.45)	27.62 (2.11)	0.151
Know behavior [mean (SD)]	41.54 (3.76)	43.07 (2.98)	0.042
Attitude behavior [mean (SD)]	56.92 (5.15)	58.93 (4.22)	0.052
Knowledge attitude behavior [mean (SD)]	62.68 (5.27)	64.89 (4.24)	0.036

Table 3 shows estimates for the association between several covariables and whether injury was caused by falling in univariate and multifactor logistic model analyses. In univariate analysis results, fathers' education level, mothers' education level, actual behavior and “Who take care children” had a significant influence on whether injured. We then built a multifactor model including gender, fathers' education level, mothers' education level, knowledge scores, attitude scores, actual behavior scores, and behavior in mind scores according to AIC and BIC (the smaller the AIC/BIC is, the better the multifactor model). According to the multi-factor logistic model, we can find attitude has positive influence on whether injured [1.13 (1.05–1.21), *P* < 0.001]. The higher of the attitude scores, the more likely the children were to be injured. Actual behavior has negative influence on whether injured [0.87 (0.78–0.97), *P* = 0.01]. The higher the attitude scores, the more likely the children were to be injured.

**Table 3:** Estimates for the association between variables and whether injured in univariate and multi-factor analysis.

		**Univariate**		**Multi-factor**	
		**OR (95%CI)**	***P***	**OR (95%CI)**	***P***
Gender	Female	ref	ref	ref	ref
	Male	0.81 (0.6–1.1)	0.18	0.81 (0.59–1.1)	0.18
Father education level	Junior high school and below	ref	ref	ref	ref
	High school/technical school/technical school	0.53 (0.22–1.31)	0.17	0.62 (0.19–1.98)	0.42
	Bachelor and above	0.87 (0.36–2.08)	0.75	0.94 (0.29–3.07)	0.92
Mother education level	Junior high school and below	ref	ref	ref	ref
	High school/technical school/technical school	0.67 (0.31–1.45)	0.31	0.75 (0.27–2.05)	0.57
	Bachelor and above	0.9 (0.42–1.93)	0.79	0.77 (0.27–2.16)	0.62
Knowledge		1.11 (0.94–1.3)	0.22	1.08 (0.91–1.28)	0.4
Attitude		1.09 (1.03–1.16)	<0.001	1.13 (1.05–1.21)	<0.001
Behavior actual		0.87 (0.78–0.96)	0.01	0.87 (0.78–0.97)	0.01
Behavior mind		0.98 (0.94–1.03)	0.46	0.95 (0.9–1)	0.07
BMI		0.98 (0.93–1.03)	0.37		
Father job	Production staff	ref	ref		
	Merchant	0.73 (0.41–1.31)	0.29		
	Institutional Staff	0.65 (0.37–1.15)	0.14		
	Professional technicians	0.77 (0.45–1.32)	0.34		
	Other	0.82 (0.46–1.47)	0.51		
Mother job	Production staff	ref	ref		
	Merchant	0.99 (0.44–2.19)	0.97		
	Institutional Staff	0.98 (0.44–2.17)	0.96		
	Professional technicians	1.37 (0.62–3.01)	0.44		
	Other	1.14 (0.52–2.48)	0.75		
Who take care	Parents	ref	ref		
	Grandparents	0.74 (0.54–1.01)	0.06		
	Relative	0.54 (0.13–2.3)	0.4		
	Nanny	0 (0-Inf)	0.97		
	Other	3.91 (0.35–43.6)	0.27		
Whether only child	Yes	ref	ref		
	No	0.8 (0.55–1.16)	0.23		

## Discussion

With total of 1,938 young children and its parents participating, this is the first study to explore the changes in KAP before and after the intervention using the government-led child injury questionnaire and to try to summarize the effect of the intervention on early children's falls. In addition, this is the first study of fall intervention focus on children aged 0–6 years. Previous study has included 122 schoolchildren between 10–12 years to apply the Safe Fall program in Physical Education classes, which can help making falls safer, diminishing the risk and severity of the injuries they cause (13). There is inadequate research on fall intervention for preschool age group. China has the largest population in the world, with young children accounting for ~5% of the total Chinese population, so it is necessary to select children and their guardians (14). As far as we know, falls are the second leading cause of unintentional injuries among children. Hospitalization events caused by falls in children can easily affect their growth and development and even affect their future development status. This study provides a positive outcome that children and their families will benefit from.

In our study, in the three KAP scores, the attitudes and behavior scores were significantly higher after the intervention than before the intervention (Figure 2), indicating that education on children's falls was effective. Education has a great influence on children. Many previous studies on educational intervention have found that appropriate education plays an important role in improving children's behavior (15–17). Unfortunately, there are few previous studies on the KAP model of childhood fall. We also observed no difference in KAP scores between minor falls and moderate to severe falls. This may indicate that educational intervention cannot improve the severity of childhood fall. Studies have shown that falls can lead to a variety of serious injuries, depending on the child's age, posture, and fall surface (12). For example, falls in infants are at a higher risk of head injuries, mainly because of reduced mobility compared to older children and a heavier head than other parts of the body (18). The average age of the subjects in this study was 2.95 years old. Educational intervention at this stage is important, since the younger the children, the more serious injuries they may have due to falls. Besides, the earlier the children and their parents receive education, the better effects that the education may incur. Children at this stage are in the stage of mother care, full of curiosity about external things, easy to have falls injury. Studies have shown that children with mothers and nurses as witnesses have a high incidence of falls, which is higher than the norm for child falls (19), suggesting that there are unobserved child falls. A British study also shows that having no stair doors in a home environment increased the risk of falls in young children by 2.5 times (20). Because children at this stage lack the ability to take care of themselves, they are at greater risks of falling, especially after the age of 1 and older has learned to walk.

In addition, univariate and paired logistic models of this study did not find that parents' educational background and family structure had an impact on injury. Although parents' educational level may influence children's fall risk through external factors such as social status and economic conditions (21, 22), our study was conducted in two neighborhoods in Shanghai with similar economic conditions, and the influence of family background may be very low. In a multifactorial analysis, parents with higher attitude scores had a higher risk of falls for their children, seemingly out of line. Past research has always suggested that attitudes trigger behavioral changes, for example, by reporting information about the benefits of vaccines that might prompt people to think they are essential. Therefore, it may increase the likelihood that people will choose to receive the vaccine (23). However, researchers say there is considerable variability in the model of attitudes influencing behavior (24). Confident and assertive attitudes are better predictors of behavior than skeptical attitudes. Similarly, attitudes based on direct experience are more likely to promote the consistency of attitudes and behaviors than those based on indirect experience, and attitudes that are easy to recall are more likely to predict behaviors than those that are hard to recall (25). Therefore, the consistency between attitude and fall intervention behavior in children is questionable and needs to be confirmed by further research.

Children are the continuation of human beings, and the prevention of children's falls is of great significance not only to the children but also to their guardians. In educational intervention, we use many new educational methods. For example, we used new media to spread health ideas. Since Chinese people widely use WeChat as an online social software, we combined the popular science content of fall prevention with network technology and distributed it to participating users through the WeChat platform, which could not only realize the educational purpose but also expand the spread of the health education concept, being a novel approach of primary prevention.

However, there are still limitations in the present paper. First, although we conducted community seminars as the intervention, we could not guarantee everyone would participate or receive the information we intended to deliver. For example, participants might be absent-minded or use their phones during the seminar. We tried to improve presence rate by reminding participants through text message before the training and asking them to sign on-side. At the end of the training, participants were asked to fill out feedback questionnaires and comment on the content of the intervention to improve the participation rate in the next activity. Another potential threat will be multiple treatment effects while the participants were under intervention, they might be also receiving other fall prevention interventions that we didn't know about. The treatment effect brought by the other situation may also have an impact on our results. Second, the social desirability bias cannot be completely avoided. It means that a parent may answer a questionnaire in a way that he or she thinks is socially acceptable, rather than honestly. This also showed up in our research, revealing differences in attitudes and behaviors. Third, the two groups of people before and after the investigation were different. We reduced this problem through pairing and randomization, but the existence of bias could not be avoided. In research that compares differences among groups, a key question is whether the groups that are compared are equivalent in all respects prior to the introduction of the independent variable or variables. Using the randomized experimental approach, equivalence is approximated through random assignment of participants to groups.

## Conclusion

The purpose of this study was to explore the effect of educational intervention on students and parents of two kindergartens in Shanghai using the KAP model and to help participants improve risk awareness and reduce the risk of childhood fall. The results showed that education was effective in preventing children from falling down, and improving the level of education will be of great help to the country and society. Our educational interventions will inform education authorities and policy makers to promote public health primary prevention of fall protection in children.

## Data Availability Statement

The original contributions presented in the study are included in the article/Supplementary Material, further inquiries can be directed to the corresponding author/s.

## Ethics Statement

The studies involving human participants were reviewed and approved by Shanghai Pudong New District Center for Disease Control and Prevention. Written informed consent to participate in this study was provided by the participants' legal guardian/next of kin.

## Author Contributions

W-YL, T-HT, YZ, DG, and HC conducted the study and drafted the manuscript. W-YL, T-HT, and DG participated in the design of the study and performed data synthesis. W-YL, YZ, and HC conceived the study and participated in its design and coordination. All of the authors read and approved the final manuscript.

## Funding

This study was supported by Excellent Youth Training Program of Health Bureau of Shanghai Pudong New Area [PWRq2021-02], Excellent Youth Training Program of Shanghai Pudong New Area Center for Disease Control and Prevention [PDCDC-XX2021-02], Youth Science and Technology Program of Health Bureau of Shanghai Pudong New Area [PW2014B-1], Academic Leaders Training Program of Pudong Health Bureau of Shanghai (Grant No. PWRd2019-11 to YZ), and the Academic Leaders Training Program of Shanghai Health Bureau (Grant No. GWV-10.2-XD24 to YZ).

## Conflict of Interest

The authors declare that the research was conducted in the absence of any commercial or financial relationships that could be construed as a potential conflict of interest.

## Publisher's Note

All claims expressed in this article are solely those of the authors and do not necessarily represent those of their affiliated organizations, or those of the publisher, the editors and the reviewers. Any product that may be evaluated in this article, or claim that may be made by its manufacturer, is not guaranteed or endorsed by the publisher.
