# Supplementary material for: The Relationship Between Knowledge, Attitude, Practice, and Fall Prevention for Childhood in Shanghai, China
Source: Front Public Health. 2022 Mar 4;10:848122. doi: 10.3389/fpubh.2022.848122 (PMC8963735; doi:10.3389/fpubh.2022.848122)
Supplement: Supplementary file 1 [file Table_1.DOCX]

| **PART 1. Basic information** | | |
| --- | --- | --- |
| **D1** | The child’s date of birth | □□□□ year □□ month □□ day |
|  | For the date of birth, fill in the year, month, and day. The date of birth in the Gregorian calendar shall prevail. If the month and/or day cannot be remembered, fill in "-9" in the corresponding column. | |
| **D2** | The child’s sex | 1 male 2 female |
| **D3** | The child’s height | cm |
| **D4** | The child’s weight | Kg |
| **D5** | The child’s household registration | 1 Shanghai household registration 2 Non-Shanghai household registration (have been living in Shanghai for years) |
| **D6** | Occupation of the child's father | 1 Agriculture, forestry, animal husbandry, fishery and water conservancy production  2 Production and transportation equipment operators and related industry  3 Commercial and service industry  4 State agencies, party organizations, enterprises, and public institutions  5 Office staff and related personnel 6 Professional technical personnel  7 Soldiers 8 Other workers  9 Students 10 Unemployed  11 Household workers 12 Retirees  13 Unclear |
|  | Including active and unemployed personnel. For employees who are engaged in several occupations (part-time) at the same time, the occupations whose working hours are the most fixed and whose income is the main source of income are the occupations of the survey. For those who work again after retiring, if the working time exceeds 1 year, they are counted as active employees and calculated based on their current occupation.  Employees ：   1. **Agriculture, forestry, animal husbandry, fishery and water conservancy production：** Personnel engaged in the production, management, and initial processing of products in agriculture, forestry, animal husbandry, fishery and water conservancy. 2. **Production and transportation equipment operators and related industry：** Personnel engaged in mineral exploration, mining, product manufacturing, engineering construction and operation of transportation equipment. 3. **Commercial and service industry：** Personnel engaged in services such as commerce, catering, tourism and entertainment, transportation, medical assistance, and social and residential life. 4. **State agencies, party organizations, enterprises, and public institutions：** In the Central Committee of the Communist Party of Shanghai and local party organizations at all levels, the Standing Committee of the People’s Congress at all levels, the People’s Political Consultative Conference, the People’s Court, the People’s Procuratorate, the State Administration, various democratic parties, trade unions, the Communist Youth League, the Women’s Federation and other people’s organizations, and mass autonomous organizations Persons who hold leadership positions and have decision-making and management powers in other social organizations and their working institutions, enterprises and institutions. 5. **Office staff and related personnel：** Persons engaged in administrative business and administrative affairs in state agencies, party organizations, enterprises, and institutions, and personnel engaged in security, fire protection, post and telecommunications, etc. 6. **Professional technical personnel：** Personnel engaged in scientific research and professional technical work. Including scientific research personnel, scientific and technological management and auxiliary personnel, aircraft and ship technicians, medical and health personnel, legal personnel, economic management professionals, teachers, teaching auxiliary personnel, art and sports personnel. 7. **Soldiers：** Soldiers serving in the military and armed police forces. 8. **Other workers：** Other practitioners who are difficult to classify.   Unemployed：   1. **Students：**Students matriculated in college or middle school. 2. **Unemployed：**Unemployed personnel or students who have yet found a job after graduation. Excluding retirees. 3. **Household worker：**Personnel mainly engaged in housework activities, such as laundry, cooking, etc., such as housewives, laid-off workers doing housework at home are unemployed. 4. **Retirees：**Refers to those who have left their jobs in accordance with national regulations and have no longer engaged in regular occupations. | |
| **D7** | Occupation of the child's mother | 1 Agriculture, forestry, animal husbandry, fishery and water conservancy production  2 Production and transportation equipment operators and related industry  3 Commercial and service industry  4 State agencies, party organizations, enterprises, and public institutions  5 Office staff and related personnel 6 Professional technical personnel  7 Soldiers 8 Other workers  9 Students 10 Unemployed  11 Household workers 12 Retirees  13 Unclear |
| **D8** | Education level of the child's father | 1 Did not receive formal school education  2 Did not graduate from elementary school  3 Elementary school 4 Middle school  5 High school/secondary school/vocational school 6 Junior college  7 Bachelor’s degree 8 Master’s degree or above  9 Unclear |
|  | Educational level refers to the highest degree of education obtained by the survey object in receiving education at home and abroad or the degree equivalent to the current level of education. For students who have not graduated or the survey object has graduated, it refers to the degree that has been obtained. If the survey object is a senior one Students, select the option "middle school".   - - 1. **Did not receive formal school education：**Refers to people who have never gone to school, or cannot read popular books and newspapers, or write notes.     2. **Did not graduate from elementary school：**Refers to a person who has received elementary education but has not graduated. It also includes people who can read popular books and newspapers, write notes, and meet literacy standards.     3. **Elementary school：**Refers to students who have graduated from elementary school, have not received junior high school education or above, or have received junior high school education, and are undergraduates and students in school.     4. **Middle school：**Refers to the undergraduate and school students who have graduated from junior high school and have not received high school education or above, or who have received high school education.     5. **High school/secondary school/vocational school：**Refers to graduates who have received high school education (including general high schools, vocational high schools and secondary professional schools), as well as undergraduate or college students who have received undergraduate or junior college education.     6. **Junior college：**Refers to graduates who have received higher education from junior colleges.     7. **Bachelor’s degree：**Refers to graduates who have received undergraduate higher education from national universities. The nationally recognized self-examinations, evening universities, TV universities, correspondence universities and other forms of undergraduate degree-granting universities are also in this category. As well as undergraduate or school students who have received postgraduate education.     8. **Master’s degree or above：**Refers to graduates who have received master's and doctoral education. | |
| **D9** | Education level of the child's mother | 1 Did not receive formal school education  2 Did not graduate from elementary school  3 Elementary school 4 Middle school  5 High school/secondary school/vocational school 6 Junior college  7 Bachelor’s degree 8 Master’s degree or above  9 Unclear |
| **D10** | Main care taker of the child(choose the most important one) | 1 Father 2 Mother  3 Grandparents 4 Siblings  5 Relatives 6 Nanny  7 Other |
| **D11** | Number of the child's siblings | 1 Only child 2 One  3 Two 4 Three or above |
| **D12** | Filler | 1 Child's mother 2 Child's father  3 Child's grandparents 4 Others(please specify) |

| **PART 2. Parents’ knowledge, attitudes, and behaviors about injuries** | | | |
| --- | --- | --- | --- |
| **Knowledge** | | | |
| K1 | Have you heard of accidental injuries? | | 1 Yes 2 No |
| K2 | Do you think accidental injuries have a great impact on children? | | 1 Yes 2 No |
| K3 | Do you think accidental injuries can be prevented and avoided? | | 1 Yes 2 No |
| K4 | Do you think accidental injuries can cause permanent disabilities? | | 1 Yes 2 No |
| K5 | What do you think is the most common accidental injury among children? (multiple choices) | | 1 Fall 2 Burns  3 Scalds 4 Animal bites  5 Suicide or homicide 6 Car accident  7 Drowning 8 Pesticides (drug poisoning)  9 Blast wounds |
| K6 | Which of the following do you think is the most important cause of death and injury among children aged 1-6 in our country? | | 1 Diseases 2 Injuries  3 Violence or abandonment  4 Congenital health problems, such as heart disease |
| K7 | Which of the following sentences do you most agree with? | | 1 Boys and girls have the same chance of being injured when they participate in the same activity  2 When participating in the same activity, boys are more likely to be injured than girls  3 Girls are more likely to be injured than boys when participating in the same activity |
| **Attitudes** | | | |
| The "never", "rarely", "sometimes", and "always" in the questionnaire options are explained as follows:  "Never" means never happens; "rarely" means 1 to 2 times out of 10;  "Sometimes" means 3 to 5 times out of 10 times; "Always" means 6 to 10 times out of 10 times. | | | |
| A1 | | Do you think it is necessary to conduct various safety education for children frequently? | 1 Not necessary 2 Optional  3 Necessary 4 Very necessary |
| A2 | | Do you think it is necessary to wear a helmet for children when skating or skateboarding? | 1 Not necessary 2 Optional  3 Necessary 4 Very necessary |
| A3 | | Do you think it is necessary to wear shoes that fit your child? | 1 Not necessary 2 Optional  3 Necessary 4 Very necessary |
| A4 | | Are you worried about accidental injury to your child? | 1 Never 2 Rarely  3 Sometimes 4 Always |
| A5 | | When a child is playing, do you think that a fall might happen? | 1 Never 2 Rarely  3 Sometimes 4 Always |
| A6 | | In your daily life, do you realize and take measures to prevent children from falling? | 1 Never 2 Rarely  3 Sometimes 4 Always |
| A7 | | Which one of the following do you think is most useful to you? | 1 Provide more information to understand what injuries are more common in children aged 1-6  2 Provide more information to understand how to reduce the incidence of child injuries  3 Provide some product information to protect children's safety (such as corner protectors, non-slip mats, etc.)  4 Provide first aid training opportunities |
| **Behaviors** | | | |
| P1 | | Are there safety locks or window shutters on the windows of your home? | 1 Yes 2 No |
| P2 | | Are there any climbing tables, chairs, sofas and other furniture beside the windows in your home? | 1 Yes 2 No |
| P3 | | Does your balcony have guardrails? | 1 Yes（If there are guardrails, the guardrails are arranged in_______ (horizontal/vertical) direction; the distance between guardrails is approximately_______ cm）  2 No |
| P4 | | Are there any climbing tables, chairs or sofas on the balcony of your home? | 1 Yes 2 No |
| P5 | | Do you protect the sharp edges and corners of your furniture? | 1 Yes 2 No |
| P6 | | Are there any anti-skid measures on wet and slippery floors such as bathrooms in the home? | 1 Yes 2 No |
| P7 | | Are there handrails on the stairs in the home? | 1 Yes（If there are guardrails, the guardrails are arranged in_______ (horizontal/vertical) direction; the distance between guardrails is approximately_______ cm）; there are no stairs  2 No |
| P8 | | When your child is learning to walk, did you choose to use a walker? | 1 Yes 2 No |
| P9 | | Would you leave your children alone on chairs, beds, and tables? | 1 Always 2 Often  3 Sometimes 4 Rarely  5 Never |
| P10 | | Will you leave your children alone at home? | 1 Always 2 Often  3 Sometimes 4 Rarely  5 Never |
| P11 | | Will you wipe it dry when there is water on the floor at home? | 1 Never 2 Rarely  3 Sometimes 4 Often  5 Always |
| P12 | | Will you pick up a towed power strip at home? | 1 Never 2 Rarely  3 Sometimes 4 Often  5 Always |
| P13 | | Would you teach your children not to climb on balconies or windows, and stop similar actions in time? | 1 Never 2 Rarely  3 Sometimes 4 Often  5 Always |
| P14 | | There are various children's entertainment facilities in the community. Would you let your children play with the children of relatives and friends without parental care? | 1 Always 2 Often  3 Sometimes 4 Rarely  5 Never |
| P15 | | Do you let your children play with the adult fitness equipment in the community? | 1 Always 2 Often  3 Sometimes 4 Rarely  5 Never |

| No. | **Question** | Option |
| --- | --- | --- |
| **F14** | **The severity of the child's injury** | 1 Mild (no obvious or slight injury)  2 Moderate (specialized treatment is required, including fracture treatment and suture)  3 Severe (need immediate emergency medical treatment or surgical treatment) |
|  | **1 Mild:** No obvious or slight injury, or just superficial abrasion, or slight cut.  **2 Moderate:** Requires specialized treatment, including fractures, or requires sutures.  **3 Severe:** Immediate emergency or surgical treatment is required, including internal hemorrhage, penetrating organ injury, and damage to large blood vessels.  4 Unclear: The severity cannot be determined. | |
